# Nitrogen Monoxide Releasing Nitric Ester Derivatives of Ibuprofen and Naproxen as COX Inhibitors, Anti-Inflammatory and Hypolipidemic Compounds

**DOI:** 10.3390/molecules30183744

**Published:** 2025-09-15

**Authors:** Paraskevi Tziona, Panagiotis Theodosis-Nobelos, Dimitris Lepesiotis, Antonis Gavalas, Eleni A. Rekka

**Affiliations:** 1 Department of Pharmaceutical Chemistry, School of Pharmacy, Aristotelian University of Thessaloniki, 54124 Thessaloniki, Greece; tzionap@gmail.com (P.T.); dlepesio@pharm.auth.gr (D.L.); agavalas@pharm.auth.gr (A.G.); 2 Department of Pharmacy, School of Health Sciences, Frederick University, Nicosia 1036, Cyprus; hsc.np@frederick.ac.cy

**Keywords:** ibuprofen, naproxen, anti-inflammatory activity, cyclooxygenase inhibition, NO release, hypolipidemic activity, COX-2 binding

## Abstract

Nitric esters are among the compounds that can liberate nitrogen monoxide (NO) in the organism. Due to the vasodilatation caused by nitrogen monoxide, NO-donors have been shown to protect endothelial function, acting as vasodilators, promoting efficient oxygen supply to tissues, to lower blood pressure, and to inhibit platelet aggregation. Incorporation of a NO-liberating moiety in the structure of non-steroidal anti-inflammatory drugs results in anti-inflammatory agents that are safer for the gastrointestinal system. In this research, ibuprofen and naproxen, two commonly applied non-steroidal anti-inflammatory drugs (NSAID), non-selective inhibitors of cyclooxygenases, were used to design novel anti-inflammatory agents able to release NO in the organism. Thus, the NSAIDs were amidated with beta-alanine and L-proline, which were able to incorporate the 2-nitro-oxyethyl moiety as the NO donor. The resulting compounds were anti-inflammatory agents, found to be more potent than the mother drugs, demonstrating remarkable inhibition of cyclooxygenase-2 over cyclooxygenase-1 and the ability to release NO in vitro. Furthermore, two of the most active anti-inflammatory compounds proved to be effective hypolipidemic agents, decreasing plasma total cholesterol, triglycerides, and LDL-cholesterol in hyperlipidemic rats significantly. The most effective compound in all the above tests was the ibuprofen derivative 5, which inhibited COX-2 by 95%, decreased inflammation by 73%, and reduced all lipidemic indices by more than 50%. Furthermore, docking experiments of compound 5 on the active sites of COX-1 and COX-2 showed that it interacts intensely with the binding site of COX-2, and the binding energy is equivalent to that of the relevant to celecoxib selective COX-2 inhibitor 4-[5-(4-bromophenyl)-3-(trifluoromethyl)-1H-pyrazol-1-yl] benzenesulfonamide (SC-5580). In conclusion, the performed structural modifications resulted not only in the improvement of the anti-inflammatory activity, compared with the parent NSAID, but also acquired strong hypolipidemic activity. Thus, the combination of structural characteristics resulting in a decrease in lipidemia, with possible inhibition of atherosclerosis, due to their anti-inflammatory activity and vasodilatation ability, via the liberated NO, may constitute a useful rationale for new compounds.

## 1. Introduction

Inflammation, a defensive mechanism of the organism, is a major factor for the progression of conditions such as asthma, rheumatoid arthritis, cardiovascular, neurodegenerative diseases, and cancer [1]. Non-steroidal anti-inflammatory drugs (NSAID), the non-selective cyclooxygenase (COX)-1 and-2 inhibitors and the selective COX-2 inhibitors, are broadly used drugs for the treatment of inflammation. However, they may cause serious and often life-threatening adverse reactions from the gastrointestinal, renal, and cardiovascular systems during long-term application [2].

Nitrogen monoxide (NO), an endogenous, highly diffusible, lipophilic, nitrogen-centered free radical, is a pleiotropic-signaling molecule that plays an important role in nervous, cardiovascular, and immune systems. In the gastrointestinal track, NO controls gastrointestinal blood flow as well as vascular and mucosal integrity [3]. Νitrogen monoxide donors could be used as antitumor agents, alone [4], chemically combined with conventional anticancer drugs [5,6], or incorporated in nanomaterials [7]. Due to the vasodilatation caused by nitrogen monoxide, NO-donors have been shown to protect endothelial function from ischemia–reperfusion injury [8], as well as in glaucoma and ocular hypertension [9]. Extended research has been devoted over the last years to the incorporation of a NO-liberating moiety in the structure of non-steroidal anti-inflammatory drugs, as safer anti-inflammatory agents for the gastrointestinal system [10,11,12]. Chemical moieties that can liberate NO in the organism include nitric esters (nitrooxy-esters) [10,13,14], nitrosothiols [15,16], NONOates (diazeniumdiolates) [17], and furoxan derivatives [18].

In this investigation, the synthesis of derivatives of two widely used nonspecific COX-1 and COX-2 inhibitors, ibuprofen and naproxen, is presented. These acids are amidated with beta-alanine and L-proline. In previous investigations, we have found that amidation of NSAID with amino acids enhances the anti-inflammatory activity of the parent drugs [19], with low gastrointestinal toxicity [20].

**Scheme 1:**
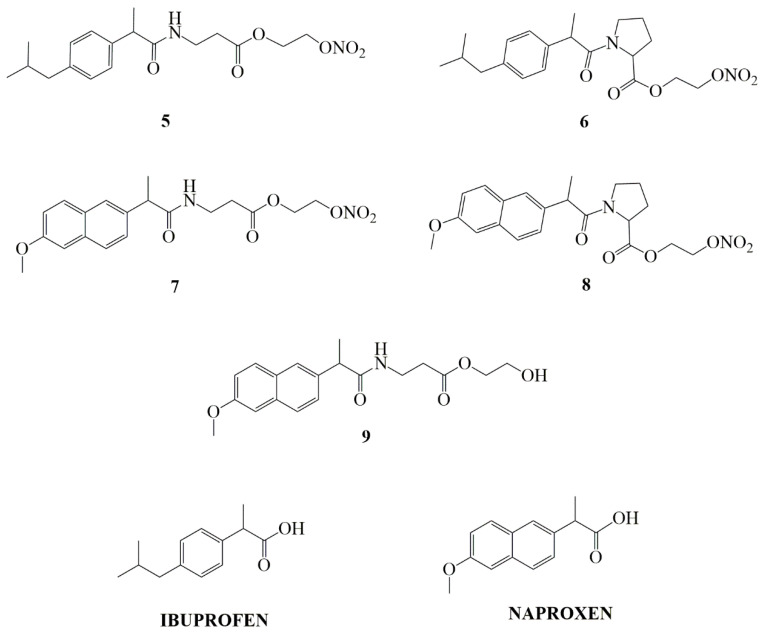
The synthesized final compounds **5–9** and the structures of ibuprofen and naproxen.

The final compounds were prepared by esterification of the amino acid carboxylic groups with 2-(nitrooxy)ethanol (compounds **5–8**, Scheme 1). Furthermore, since 2-hydroxyethyl derivatives of the NSAID are produced as hydrolysis metabolites of the corresponding nitric ester, the 2-hydroxyethyl derivative **9** of **7** is synthesized and tested for its anti-inflammatory activity. These compounds were tested in vitro and in vivo. The in vitro experiments included determination of COX-1, COX-2, and lipoxygenase inhibition as well as NO liberation. The ability of these compounds to reduce acute inflammation and to affect lipidemic indices in tyloxapol-induced hyperlipidemic rats was also evaluated, and molecular docking studies were performed.

## 2. Results and Discussion

### 2.1. Synthesis

Beta-alanine methyl ester hydrochloride (methyl 3-aminopropanoate hydrochloride) and *L*-proline methyl ester hydrochloride ((*S*)-methyl pyrrolidine-2-carboxylate hydrochloride) were prepared via the reaction of the acid chlorides with methanol, as previously described [19].

The NSAIDs reacted with the amino acid methyl esters in the presence of N,N′-dicyclohexylcarbodiimide (DCC) and trimethylamine, to give the respective amides. Methyl esters were hydrolyzed by a NaOH solution in water/dioxane. Subsequent acidification led to the corresponding acids, with minor differences in the reaction time and yields (68–98%) [19].

**Scheme 2:**
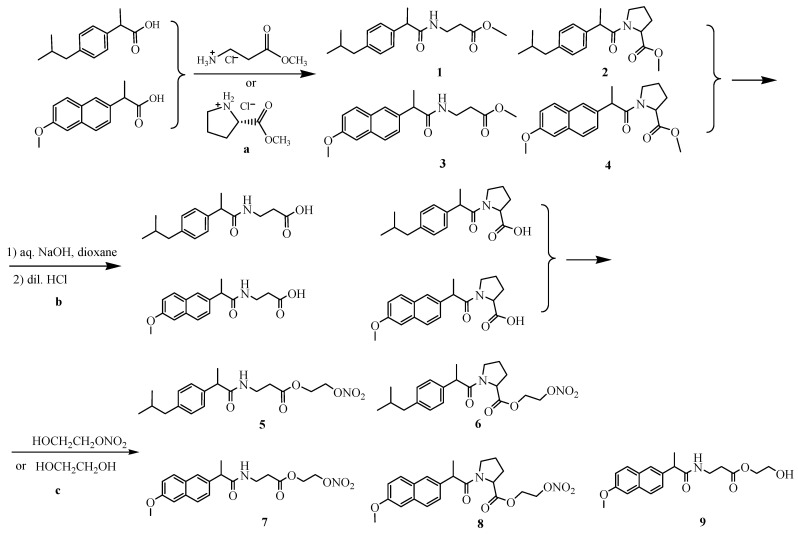
Synthetic route for the final compounds **5–9**. Reaction conditions: **a.** Amidation of the carboxylic acids, using DCC, Et_3_N, in CH_2_Cl_2_, r.t. 12 h. **b.** Alkaline ester hydrolysis, subsequent acidification to the free acids. **c.** Esterification using DCC, DMAP, in CH_2_Cl_2_, r.t., 2–4 h.

The final products were obtained from the esterification of the carboxylic group of the intermediate amides with 2-nitrooxyethanol or 1,2-dihydroxyethane, using DCC in the presence of 4-dimethylaminopyridine (DMAP), with yields of 60–86% (Scheme 2). The spectroscopic (^1^H-NMR, ^13^C-NMR) studies and chemical analysis (C, H, N) support their molecular structures.

### 2.2. In Vitro Studies

#### 2.2.1. Inhibition of Cyclooxygenase (COX)-1 and-2 Activity

Cyclooxygenases-1 and-2 catalyze the production of prostanoids from arachidonic acid. The constitutively expressed COX-1 participates in the maintenance of cellular homeostasis. In the gastrointestinal tract, it protects gastric mucosa through continuous production of cytoprotective prostaglandins, especially of PGE2 and PGI2 [21]. COX-2 is induced in response to inflammatory stimuli and is responsible for producing prostaglandins such as prostaglandin E2, the principal mediator of inflammation [22]. However, COX-2 is constitutively expressed in various organs and cells. In the kidney, it is greatly regulated in response to changes in intravascular volume [23]. Furthermore, constitutive expression of COX-2 in cardiomyocytes plays a cardioprotective role [24]. COX-2 is constitutively expressed in the brain [25] and the pancreatic islet cells [26].

The effect of the synthesized compounds, as well as that of ibuprofen and naproxen, used as reference compounds, (concentration 0.05 μM) on both COX isoforms, was examined. The concentration of the substrate, arachidonic acid, was 0.1 μM. Results are shown in Table 1.

All nitric esters offered lower or similar inhibition of COX-1 activity, compared with the parent NSAID, with the exception of **8**, which presented a remarkable inhibitory activity on COX-1. Very significant inhibition of COX-2 was observed with **5**, which was twice as active as ibuprofen. About the same is true, although to a lesser extent, for **7**, compared with naproxen. In addition, the ratio of COX-1/COX-2 inhibition was about the same for naproxen (2.0) and **7** (1.75). The hydroxyethyl ester **9** produced high inhibition of COX-1 isoform, with no change in the ability to inhibit COX-2 (compared to naproxen), in comparison with the nitric ester **7**, which was stronger than the parent naproxen in both isoforms. The other compounds had medium-to-low inhibitory activity on COX-2. Overall, the two ibuprofen derivatives were the most active against COX-2, while **5** was the most potent inhibitor of both COX isozymes.

#### 2.2.2. Inhibition of Lipoxygenase (LOX) Activity

Lipoxygenases (LOX) are cytosolic enzymes with a non-heme iron in their active site. They catalyze the insertion of two oxygen atoms into polyunsaturated fatty acids which contain the *cis*-1,4-pentadiene structure, forming the corresponding hydroperoxyl fatty acids. Lipoxygenases constitute the second major pathway of arachidonic acid metabolism, involved in the synthesis of leukotrienes, inflammatory mediators produced in leukocytes. Leukotrienes are implicated in various disorders, e.g., asthma, arthritis, atherosclerosis, and neurodegenerative and autoimmune diseases.

As COX inhibition may lead to upregulation of the lipoxygenase (LOX) pathway for the metabolism of arachidonic acid, parallel LOX inhibition may weaken the appearance of adverse effects [27].

5-Lipoxygenase activity contributes to atherosclerosis via oxidation of low-density lipoprotein. Furthermore, studies using 5-lipoxygenase-deficient mice show that 5-lipoxygenase activity may contribute to stress and depression behavior. Soybean lipoxygenase-1 can use arachidonic acid as substrate, with about 15% of activity for linoleic acid. Arachidonic acid binding sites in plant lipoxygenases possess almost the same similarity as animal 5-lipoxygenase [28]. Furthermore, the primary structures of soybean and human lipoxygenases possess considerable homology, especially on their active sites [29]. Thus, soybean lipoxygenase is often used for the study of anti-inflammatory agents.

The effect of the synthesized compounds and of the parent NSAIDs on lipoxygenase is presented in Table 2. The IC_50_ of nordihydroguaiaretic acid (NDGA), an antioxidant compound acting as a nonspecific inhibitor of lipoxygenase [30], is also included as a positive reference.

We applied the same procedure but using a higher concentration of linoleic acid than the saturating substrate concentration (1 mM) and we observed no inhibition. These results indicate that the examined compounds may act as competitive inhibitors of lipoxygenase, since inhibition declines by increasing substrate concentration.

Ibuprofen and naproxen demonstrated low inhibition of LOX (Table 2). Compounds incorporating the proline structure also had low effect; however, insertion of the beta-alanine moiety lead to a considerable increase in inhibition. This may be attributed to the more flexible chain of alanine, compared with proline, enabling the molecule to occupy better the U-shaped lipophilic site of the enzyme active center, which was intended for the flexible molecules of arachidonic or linoleic acid.

**Figure 1:**
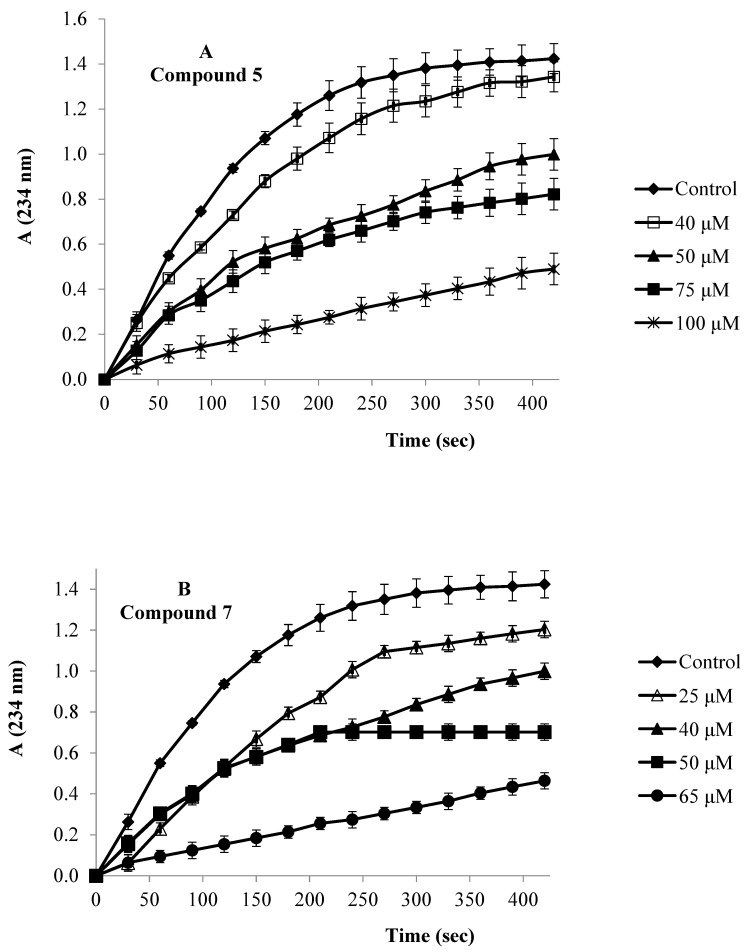
Course of LOX activity in relation to the duration of incubation, as affected by different concentrations of **5** (**A**) and **7** (**B**).

The effect of the most active compounds **5** and **7** on the progression of LOX activity is shown in Figure 1A,B.

#### 2.2.3. General Remarks on Enzyme Inhibition Assays

Concerning the effects of the compounds on the activities of both enzymes (COX and LOX) and the possible interference of the liberated NO in the assays, a possible involvement might be due to the antioxidant activity of NO, or to the interference of NO with the oxidation state of iron (heme for COX, or nonheme for LOX). Long (45 min) incubation of these compounds with Fe^2+^-ascorbic acid in the presence of microsomal membranes did not show any detectable lipid peroxidation (Section 2.2.5). 

As for the possible involvement of NO in the oxidation state of iron, a large difference is observed, particularly in COX-2 inhibition, by compounds **5** and **6**, but also between COX-1 inhibition values by them. These results mainly indicate steric effects of the molecules on COX binding site more than NO interference. Furthermore, compound **9**, without a NO-releasing capacity, still inhibits COX enzymes. Especially, COX-1 inhibition is higher than that of the nitric ester **7**. Finally, the very low free energy of binding compound **5** to COX-2 (comparable to that of the reference selective COX-2 inhibitor, Table 6) corroborates the binding of the molecule to the enzyme.

An analogous situation is observed with LOX inhibition, especially by compounds **5** and **6**, but also by **7** and **8**, which also points to steric differences rather than to a possible generation and subsequent inhibitory activity of NO.

Thus, without excluding a possible intervention of NO in the in vitro assays, it can be suggested that the observed results are largely due to the direct inhibitory activity of the compounds.

#### 2.2.4. Nitrogen Monoxide Release

Nitrogen monoxide (NO), a nitrogen-centered free radical, is produced in the organism from L-arginine, in a two-step reaction catalyzed by nitrogen oxide synthases (NOS). It acts as a vasodilator, promoting efficient oxygen supply to tissues, lowers blood pressure, and it inhibits platelet aggregation [31]. Furthermore, NO is a neurotransmitter and a mitochondrial regulator. It can act as an antioxidant, by scavenging superoxide anion radical, and as an anti-inflammatory agent.

All compounds could liberate NO, and a linear increase in the amount of released NO is observed with increasing compound concentration (Table 3).

Again, **5** was the most potent NO donor, followed by **6**. No differentiation was observed between the two naproxen derivatives (**7**, **8**).

#### 2.2.5. Effect on Lipid Peroxidation

Thermally inactivated microsomal fraction from the liver of untreated rats was used as a lipid source. The reaction of peroxidation was induced by ascorbic acid and ferrous sulfate, and the degree of peroxidation was estimated as 2-thiobarbituric acid reactive material photometrically [32].

All compounds presented minor antioxidant effects. Nitric esters may liberate nitrogen monoxide in the organism, and NO is a scavenger of superoxide anion radical. However, it seems that under the conditions of this in vitro experiment, no such activity could be expressed; thus, no antioxidant effect could be observed.

### 2.3. In Vivo Studies

#### 2.3.1. Effect on Acute Inflammation Produced by Carrageenan Administration

The carrageenan-induced paw edema is a common, reproducible, nonimmune model of acute inflammation, with predictive value for detecting the anti-inflammatory activity of compounds. The development of carrageenan inflammation involves several inflammatory mediators acting in two phases. The first phase (during the first hour post-injection) involves histamine, serotonin, bradykinin, and reactive oxygen species, while the second phase (2.5 to 6 h) implicates prostaglandins and cytokines (IL-1β, IL-6, IL-10, TNF-α) [33]. The anti-inflammatory effect of the compounds and of ibuprofen and naproxen 3.5 h after carrageenan administration is demonstrated in Table 4.

All nitric esters showed anti-inflammatory activity well above the parent NSAIDs, more than double that of ibuprofen and naproxen. The most active was **5** and this effect goes in parallel with the very strong inhibition of COX-2 (Table 1). Compound **6** is still an effective anti-inflammatory compound, although less potent COX-2 inhibitor.

The methyl esters **1** and **2** expressed low anti-inflammatory activity, compared with their nitric ester analogues or even ibuprofen [19]. Possibly, this is due to less effective interaction with COX-2, or to unfavorable pharmacokinetics. Interestingly, the 2-hydroxyethyl ester **9** demonstrated high anti-inflammatory activity, compared to naproxen, practically equipotent to **7**. Additionally, compound **9** could inhibit COX-2 almost as efficiently as **7** (Table 1). This may have a more general interest, since the described compounds may have the advantage that, after NO liberation in the organism, which also protects the gastrointestinal tract from the side effects of COX-2 inhibition, the hydrolyzed metabolites can still act as anti-inflammatory agents.

#### 2.3.2. Effect on Lipidemic Indices in Hyperlipidemic Rats

Hyperlipidemia is a serious risk factor for the development of atherosclerosis and thrombosis. Other major factors are diabetes, obesity, hypertension, and smoking. It is known that atherosclerosis is a chronic inflammation of the vasculature, triggered by the above risk factors [34]. In arteriosclerosis, low-density lipoprotein cholesterol (LDL) is oxidized to oxLDL, which is accumulated in the inner vascular wall. Macrophages engulf oxLDL, forming foam cells. oxLDL may upregulate expression of NF-κB, adhesion molecules, and other inflammatory factors leading to inflammation [35]. In addition, oxLDL disturbs NO production by NO synthase of endothelial cells (eNOS), thus facilitating the activation of inducible NOS (iNOS), which promotes vascular inflammation, while normal NO levels can prevent oxLDL formation. Since NO can down-regulate NF-κΒ, low levels of NO further contribute to inflammation. 

Cholesterol is oxidized in oxLDL, to products toxic to vascular endothelium, and thus, to NO production.

High triglyceride levels can also impair NO production and can create a prooxidant environment. In addition, NO affects lipoprotein lipase, that catalyzes triglyceride hydrolysis to free fatty acids and monoacetylglycerol.

Furthermore, inadequate NO deprives the vasculature of a vasodilating factor, which may worsen hypertension and thrombosis.

Tyloxapol (Triton WR 1339) is a nonionic liquid polymeric surfactant. When tyloxapol is administered parenterally (*i.p.* or *i.v.*), it produces experimental hyperlipidemia, mainly via inhibition of lipoprotein lipase. Hyperlipidemia increases rapidly and linearly for the first 24 h post administration, followed by a gradual decrease [36].

The two most active anti-inflammatory NO donors, compounds **5** and **6,** were evaluated for their hypolipidemic activity in hyperlipidemic rats. Simvastatin and ibuprofen were tested for comparison. Results are shown in Table 5.

An important decrease in all three lipidemic indices was observed after the administration of **5** and **6**, which seem to be equipotent. Simvastatin was better in TC and LDL reduction; however, it could not decrease triglycerides, as it is already known [37]. Ibuprofen, at the same dose (150 μmol/kg) caused a negligible effect and, at double dose (300 μmol/kg), it produced a moderate effect, still quite lower than that of the nitro-compounds.

### 2.4. In Silico Study

Docking of Compound **5** to COX-1 and COX-2

In order to further investigate the outstanding activity of **5** on cyclooxygenase and inflammation, we performed a docking-assisted prediction of binding free energy values of **5** on both COX isomers and the involved hydrogen bonds (Table 6). It can be seen that the binding energy of **5** to COX-1 is higher than that of ibuprofen, whereas the binding energy to COX-2 is as low as that of SC-558, a selective inhibitor of COX-2 structurally related to celecoxib. 

**Figure 2:**
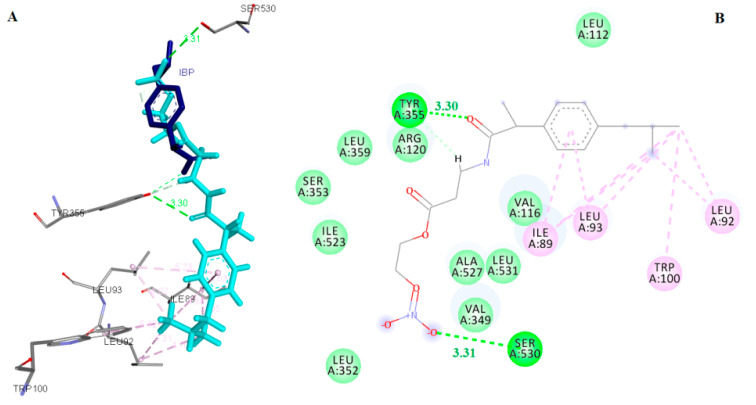
(**A**). Docked compound **5** (cyan) superimposed on the co-crystallized ibuprofen (blue) in the binding pocket of COX-1. (**B**) 2D diagram of compound **5** in the binding pocket of COX-1. Hydrogen bonds are shown as green dotted lines.

**Figure 3:**
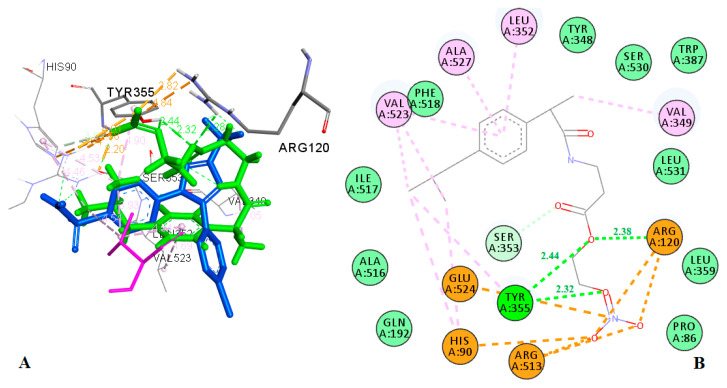
(**A**). Docked compound **5** (green) superimposed on the co-crystallized SC-558 (blue) in the binding pocket of COX-2. (**B**) 2D diagram of compound **5** in the binding pocket of COX-2. Hydrogen bonds are shown as green dotted lines. (**B**): pink = π-π, π-alkyl, light green: vdW, orange: electrostatic, dark green: H-bond.

Docked compound **5** on the active site of COX-1 and COX-2 is shown in Figure 2 and Figure 3. 

## 3. Materials and Methods

### 3.1. General

All commercially available materials were obtained from Merck (Kenilworth, NJ, USA) or Sigma (St. Louis, MO, USA) and used without further purification. The ^1^H NMR and ^13^C-NMR spectra were recorded using an AGILENT DD2-500 MHz (Santa Clara, CA, USA) spectrometer. All chemical shifts are reported in *δ* (ppm), and signals are designated as follows: s, singlet; d, doublet; t, triplet; m, multiplet. Melting points (m.p.) were determined with a MEL-TEMPII (Laboratory Devices, Sigma-Aldrich, Milwaukee, WI, USA) apparatus and were uncorrected. The microanalyses were performed on a Perkin-Elmer 2400 CHN elemental analyzer (Waltham, MA, USA). Thin-layer chromatography (TLC silica gel 60 F254 aluminum sheets, Merck (Kenilworth, NJ, USA)) were used to monitor the reactions, and the spots were visualized under UV light. UV–visible determinations were performed using a Shimadzu UV-1700 Pharma Spec spectrophotometer (Kyoto, Japan).

κ-Carrageenan and lipoxygenase type I-B from soybean were supplied by Sigma (St. Louis, MO, USA). COX inhibition was estimated using the “COX Inhibitor Screening Assay” kit (Cayman Chemical Co., Ann Arbor, MI, USA). For the determination of plasma cholesterol, triglyceride, and LDL-cholesterol concentrations, SPINREACT S.A. Cholesterol-LQ, Triglycerides-LQ, and LDL-Cholesterol D kits were used (Ctra. Santa Coloma, Spain).

Adult Wistar male and female rats (160–220 g, 3–4 months old), for the in vivo experiments, were kept in the Centre of the School of Veterinary Medicine (EL54 BIO42), Aristotelian University of Thessaloniki, which is registered by the official state veterinary authorities, and all procedures were performed in harmonization with the European Directive 2010/63/EEC. The experimental protocols were approved by the Animal Ethics Committee of the Prefecture of Central Macedonia (no. 270073/2499 and 270079/2500). Rats were housed in controlled rooms, humidity 50–60%, temperature 23 °C, with a 12 h light/dark cycle, and free access to standard laboratory chow and tap water.

### 3.2. Synthesis

#### 3.2.1. Synthesis of Intermediate Compounds

The methyl esters of beta-alanine and *L*-proline, as well as their amides with ibuprofen and naproxen (compounds **1–4**) were prepared and characterized according to our previous report [20]. 2-Nitrooxy-ethanol was synthesized from ethane-1,2-diol, as described [10].

#### 3.2.2. General Method for the Synthesis of the Final Compounds **5**–**9**:

The respective acid (1 mmol, ibuprofen was racemate, naproxen was the *S*-enantiomer) and DCC (1 mmol) were dissolved in dry dichloromethane and mixed. Then, 2-nitrooxy-ethanol (1.1 mmol) or 1,2-ethane-1,2-diol (4 mmol) and 4-dimethylaminopyridine (DMAP, 0.1 mmol), all dissolved in dry dichloromethane, were added and stirred at ambient temperature for 2–4 h. The reaction mixture was filtered, and the filtrate was washed with water, dried (Na_2_SO_4_), and concentrated under reduced pressure. The residue was purified with flash chromatography, using petroleum ether/ethyl acetate as eluent.

2-(Nitrooxy)ethyl-3-(2-(4-isobutylphenyl)propanamido)propanoate (**5**).

Flash column chromatography (petroleum ether/ethyl acetate, 8/1). Viscous liquid, yield 67%.

^1^H NMR (CDCl_3_), *δ* (ppm): 0.89 [d, 6H, *J* = 6.6 Hz, (C**H_3_**)**_2_**CHCH_2_-], 1.48 [d, 3H, *J* = 7.2 Hz, -ph-CH(C**H_3_**)CO-], 1.88–1.80 [m, 1H, (CH_3_)_2_C**H**CH_2_-], 2.44 [d, 2H, *J* = 7.2 Hz, (CH_3_)_2_CHC**H_2_**-], 2.51 [td, 2H, *J* = 5.9, 2.3 Hz, -NHCH_2_C**H_2_**-], 3.47–3.40 [m, 2H, -NHC**H_2_**CH_2_-], 3.49 [q, 1H, *J* = 7.2 Hz, -phC**H**(CH_3_)CO-], 4.33–4.26 [m, 2H, -OCH_2_C**H_2_**ONO_2_], 4.60–4.58 [dd, 2H, *J* = 11.2, 6.6 Hz, -OC**H_2_**CH_2_ONO_2_], 5.86 [bs, 1H, -N**H**CH_2_CH_2_-], 7.10 [d, 2H, *J* = 8.0 Hz, phenyl C3, C5], 7.16 [d, 2H, *J* = 8.0 Hz, phenyl C2, C6]. 

^13^C NMR (CDCl_3_), *δ* (ppm): 18.31 [1C, CH**C**H_3_CONH-], 22.34 [2C, (**C**H_3_)_2_CHCH_2_-], 30.11 [1C, (CH_3_)_2_**C**HCH_2_-], 33.73 [1C, -NHCH_2_**C**H_2_-], 34.98 [1C, -NH**C**H_2_CH_2_], 44.98 [1C, -ph-**C**H(CH_3_)CO-], 46.61 (CH_3_)_2_CH**C**H_2_-], 60.29 [1C, *J* = 7.1 Hz O**C**H_2_CH_2_ONO_2_-], 70.14 [1C, OCH_2_**C**H_2_ONO_2_-], 127.24 [2C, phenyl C2, C6], 129.59 [2C, phenyl C3, C5], 138.06 [1C, phenyl C1], 140.77 [1C, phenyl C4], 175.15 [1C, O-**C**O-], 176.41 [1C, N-**C**O-]. 

Anal. Calculated for C_18_H_26_N_2_O_6_ (%): C 59.00, H 7.15, N 7.65. Found: C 59.03, H 7.12, N 7.36.

2-(Nitrooxy)ethyl-1-(2-(4-isobutylphenyl)propanoyl)pyrrolidine-2-carboxylate (**6**).

Flash column chromatography (petroleum ether/ethyl acetate, 10/1). Viscous liquid, yield 65%. 

^1^H NMR (CDCl_3_), *δ* (ppm): 0.90–0.87 [m, 6H, (C**H_3_**)_2_CHCH_2_-], 1.42, 1.41 [d, 3H, *J* = 7.1 Hz, -ph-CH(C**H_3_**)CO-], 1.86–1.78 [m, 2H, CH_2_C**H_2_**CH_2_CHCO-], 1.96–1.87 [m, 2H, CH_2_CH_2_C**H_2_**CHCO-], 2.11–1.96 [m, 1H, (CH_3_)_2_C**H**CH_2_-], 2.43 [dd, 2H, *J* = 7.1, 3.8 Hz, (CH_3_)_2_CHC**H_2_**-], 3.29–3.20 [m, 1H, C**H_2_**CH_2_CH_2_CHCO-], 3.55–3.45 [m, 1H, C**H_2_**CH_2_CH_2_CHCO-], 3.79–3.63 [m, 1H, -ph-C**H**(CH_3_)CO-], 4.75–4.33 [m, 5H, -CHCH_2_CH**_2_**C**H**CO-, -OHCH_2_C**H_2_**-, -OHC**H_2_**CH_2_-], 7.19–7.03 [m, 4H, phenyl C3, C5, phenyl C2, C6].

^13^C NMR (CDCl_3_), *δ* (ppm): 20.14, 20.02 [1C, ph-CH(**C**H_3_)CO-], 22.40, 22.37 [2C, (**C**H_3_)_2_CHCH_2_-], 25.05, 24.85 [1C, CH_2_**C**H_2_CH_2_CHCO-], 28.96 [1C, (CH_3_)_2_**C**HCH_2_-], 30.14, 30.10 [1C, CH_2_CH_2_C**H_2_**CHCO-], 44.43, 44.41 [1C, -ph-C**H**(CH_3_)CO-], 45.01, 45.00 (CH_3_)_2_CH**C**H_2_-], 46.79 [1C, **C**H**_2_**CH_2_CH_2_CHCO-], 59.03, 59.01 [1C, -OHC**H_2_**CH_2_O-], 60.66, 60.51 [1C, -CHCH_2_CH**_2_**C**H**CO-], 70.42, 70.35 [1C OHCH_2_**C**H_2_O-], 127.22, 127.18 [2C, phenyl C2, C6], 129.48, 129.38 [2C, phenyl C3, C5], 138.24, 138.14 [1C, phenyl C1], 140.31, 140.18 [1C, phenyl C4], 172.19, 171.82 [1C, O-**C**O-], 172.95, 172.72 [1C, N-**C**O-].

Anal. Calculated for C_20_H_28_N_2_O_6_ (%): C 61.21, H 7.19, N 7.14. Found: C 61.39, H 7.41, N 6.88.

2-(Nitrooxy)ethyl-3-(2-(6-methoxynaphthalen-2-yl)propanamido)propanoate (**7**).

Flash column chromatography (petroleum ether/ethyl acetate, 5/1). Viscous liquid, yield 86%.

^1^H NMR (CDCl_3_), *δ* (ppm): 1.57 [d, 3H, *J* = 7.1 Hz-CHC**H_3_**CONH-], 2.50 [t, 2H, *J* = 6.0 Hz, -NHCH_2_C**H_2_**-], 3.51–3,40 [m, 2H, -NHC**H_2_**CH_2_COO-], 3.66 [q, 1H, *J* = 7.1 Hz, -phC**H**(CH_3_)CONH-], 3.91 [s, 3H, C**H_3_**O naphthyl-], 4.22–4.11 [m, 2H, -OC**H_2_**CH_2_ONO_2_], 4.46 [t, 2H, *J* = 4.6 Hz, -OCH_2_C**H_2_**ONO_2_], 5.86 [s, 1H, -CON**H**CH], 7.11 [d, 1H, *J* = 2.0 Hz, napthylC5], 7.15 [dd, 1H, *J* = 8.9, 2.4 Hz, napthylC7], 7.35 [dd, 1H, *J* = 8.5, 2.0 Hz, napthylC3], 7.64 [s, 1H, napthylC1], 7.70 [2H, dd, *J* = 8.5, 2.7 Hz, napthylC8, C4].

^13^C NMR (CDCl_3_), *δ* (ppm): 18.30 [1C, -CH**C**H_3_CONH-], 33.77 [1C, -NHCH_2_**C**H_2_-], 34.95 [1C, -NH**C**H_2_CH_2_-], 46.98 [1C, -ph**C**H(CH_3_)CONH-], 55.31 [1C, **C**H_3_O naphthyl-], 60.18 [1C, *J* = 7.1 Hz O**C**H_2_CH_2_ONO_2_-], 70.05 [1C, OCH_2_**C**H_2_ONO_2_-], 105.58 [1C, napthylC5], 119.14 [1C, napthylC7], 126.02 [1C, napthylC1], 126.14 [1C, napthylC8’], 127.48 [1C, napthylC4], 128.94 [1C, napthylC3], 129.18 [1C, napthylC8], 133.69 [1C, napthylC4’], 136.40 [1C, napthylC2], 157.73 [1C, napthylC6], 171.82 [1C, -O-**C**=O], 174.36 [1C, -N-**C**=O].

Anal. Calculated for C_19_H_22_N_2_O_7_ (%): C 58.46, H 5.68, N 7.18. Found: C 58.68, H 5.46, N 7.19.

2-(Nitrooxy)ethyl-1-(2-(6-methoxynaphthalen-2-yl)propanoyl)pyrrolidine-2-carboxylate (**8**).

Flash column chromatography (petroleum ether/ethyl acetate, 5/1). Viscous liquid, yield 77%.

^1^H NMR (CDCl_3_), *δ* (ppm): 1.49 [d, 3H, *J* = 6.9 Hz, -CHC**H_3_**CONH-], 1.91–1.79 [m, 3H, -NCH_2_C**H_2_**CH_2_CH και -NCH_2_CH_2_C**H**_2_CH], 2.23–2.12 [m, 1H, -NCH_2_CH_2_C**H**_2_CH], 3.59–3.49, 3.30–3.21 [m, 2H, -NC**H_2_**CH_2_CH_2_CH], 3.91 [s, 3H, C**H_3_**O naphthyl-], 4.13–4.11 [m, 1H, -C**H**CH_3_CONH-], 4.37 [t, 2H, *J* = 4.6 Hz, -OCH_2_C**H_2_**ONO_2_], 4.62–4.48 [m, 3H, -OC**H_2_**CH_2_ONO_2_ και -NCH_2_CH_2_CH_2_C**H**], 7.11 [s, 1H, napthylC5], 7.14 [dd, 1H, *J* = 8.5, 2.5 Hz, napthylC7], 7.38 [dd, 1H, *J* = 8.5, 1.3 napthylC1], 7.67 [s, 1H, napthylC8], 7.73–7.68 [m, 2H, *J* = 8.5 Hz, napthylC3, C4].

^13^C NMR (CDCl_3_), *δ* (ppm): 20.11, 20.03 [1C, -CH**C**H_3_CONH-], 24.95 [1C, -NCH_2_**C**H_2_CH_2_CH], 28.97 [1C, -NCH_2_**C**H_2_CH_2_CH], 44.86, 44.70 [1C, **C**HCH_3_CONH], 46.88, 46.76 [1C, -N**C**H_2_CH_2_CH_2_CH], 55.41, 55.23 [1C, **C**H_3_O naphthyl-], 59.03, 59.00 [1C, -O**C**H_2_CH_2_ONO_2_], 60.49 [1C, -NCH_2_CH_2_CH_2_C**H**], 70.21, 70.24 [1C, -OCH_2_**C**H_2_ONO_2_], 105.72, 105.51 [1C, napthylC5], 119.04, 118.85 [1C, napthylC7], 126.18, 125.99 [1C, napthylC1], 126.42 [1C, napthylC8’], 127.37, 127.21 [1C, napthylC4], 129.09, 129.03 [1C, napthylC3], 129.25 [1C, napthylC8], 133.47 [1C, napthylC4’], 136.09 [1C, napthylC2], 157.57 [1C, napthylC6], 171.75 [1C, -O-**C**=O], 172.58 [1C, -N-**C**=O].

Anal. Calculated for C_21_H_24_N_2_O_7_ (%): C 60.57, H 5.81, N 6.73. Found: C 60.59, H 6.11, N 7.11.

2-Hydroxyethyl 3-(2-(6-methoxynaphthalen-2-yl)propanamido)propanoate (**9**).

Flash column chromatography (petroleum ether/ethyl acetate, 1/5). White solid, mp. 56 °C, yield 32%.

^1^H NMR (CDCl_3_), *δ* (ppm): 1.55 [d, 3H, *J* = 7.1 Hz -CHC**H_3_**CONH-], 2.08 [s, 1H, -OCH_2_CH_2_O**H**], 2.45 [t, 2H, *J* = 6.0 Hz, -NHCH_2_C**H_2_**-], 3.46 [q, 2H, *J* = 6.0 Hz, -NHC**H_2_**CH_2_COO-], 3.68–3.62 [m, 3H, -phC**H**(CH_3_)CONH- and -OCH_2_C**H_2_**OH], 3.89 [s, 3H, C**H_3_**O naphthyl-], 4.10–4.03 [m, 2H, -OC**H_2_**CH_2_OH], 6.12 [s, 1H, -CON**H**CH], 7.10 [bs, 1H, *J* = 2.0 Hz, napthylC5], 7.13 [dd, 1H, *J* = 8.9, 2.4 Hz, napthylC7], 7.34 [d, 1H, *J* = 8.5 Hz, napthylC3], 7.62 [s, 1H, napthylC1], 7.68 [2H, dd, *J* = 8.5, 2.5 Hz, napthylC8, C4].

^13^C NMR (CDCl_3_), *δ* (ppm): 18.36 [1C, -CH**C**H_3_CONH-], 34.44 [1C, -NHCH_2_**C**H_2_-], 35.23 [1C, -NH**C**H_2_CH_2_-], 46.91 [1C, -ph**C**H(CH_3_)CONH-], 55.30 [1C, **C**H_3_O naphthyl-], 60.59 [1C, *J* = 7.1 Hz OCH_2_**C**H_2_ONO_2_-], 66.19 [1C, O**C**H_2_CH_2_ONO_2_-], 105.64 [1C, napthylC5], 119.10 [1C, napthylC7], 126.03 [1C, napthylC1], 126.17 [1C, napthylC8’], 127.44 [1C, napthylC4], 128.93 [1C, napthylC3], 129.19 [1C, napthylC8], 133.70 [1C, napthylC4’], 136.26 [1C, napthylC2], 157.71 [1C, napthylC6], 172.15 [1C, -O-**C**=O], 174.78 [1C, -N-**C**=O].

### 3.3. Biological Experiments

#### 3.3.1. In Vitro Assays

- Inhibition of Cyclooxygenase (COX)-1 and -2 Activity

The effect of compounds on COX-1 and COX-2 activity was measured using a commercial kit and following the instructions of the manufacturer. The kit uses ovine COX-1 and human recombinant COX-2 enzymes. The assay determines PGF_2a_ produced from COX-derived PGH_2_ after reduction with SnCl_2_. The prostanoid product was evaluated via enzyme immunoassay using a broadly specific antibody that binds to all the major prostaglandin compounds [19].

- Inhibition of Lipoxygenase (LOX) Activity

The reaction mixture contained the examined compounds dissolved in absolute ethanol (analytical grade, iron content <10^−5^% *w*/*v*) soybean lipoxygenase (in saline, 250 µ/mL) and sodium linoleate (100 µM), in Tris-HCl buffer, pH 9.0. The reaction was followed for 7 min at 28 °C, recording the absorbance every 30 s at 234 nm. Nordihydroguaiaretic acid (NDGA) was used as a reference [19].

- Nitrogen Monoxide Release

The nitric acid esters **5–8** dissolved poorly in water; thus, they were dissolved in 7/3 (*v*/*v*) mixture of DMSO/water at various concentrations and incubated overnight at room temperature with cadmium. *S*-nitroso-*N*-acetylpenicillamine (SNAP) was dissolved either in DMSO/water 7/3 or in water only and treated as above.

Aliquots were taken from each sample and added to an equal volume of *N*-naphthylaminoethylamine (0.2%) and sulfanilamide (2%) solution in 3 N hydrochloric acid (Griess reagent). Nitric oxide release was estimated spectrophotometrically (540 nm). NO release from SNAP (100 μM in DMSO/water 7/3 and in water) was found to be 56.3 and 56.7 μΜ, respectively, confirming that the addition of DMSO did not influence the procedure [10].

#### 3.3.2. In Vivo Experiments

- Effect on Acute Inflammation Produced by Carrageenan Administration

The compounds under examination (in saline with a few drops of Tween 80) were injected *i.p.* (0.15 mmol/kg) into adult, healthy rats, just after the *i.d*. injection of 0.1 mL of an aqueous carrageenan solution (1% *w*/*v*) in the hind paws of rats (six rats were used for each compound and control group). The created edema, after 3.5 h, was estimated as paw weight increase [10].

- Effect on Lipidemic Indices in Hyperlipidemic Rats

A solution of Triton WR 1339 (tyloxapol) in saline was injected *i.p.* (200 mg/kg) into rats to induce hyperlipidemia. After 1h, the examined compounds (0.15 mmol/kg, suspended in saline with a few drops of Tween 80) were given *i.p* (six rats were used for each compound and control group). 24 h later, blood was taken from the aorta in heparinized tubes and used for the determination of plasma total cholesterol (TC), triglyceride (TG), and low-density lipoprotein cholesterol (LDL-C) concentrations, using commercial kits, against standard solutions [32].

### 3.4. Molecular Docking Studies

Molecular modeling studies were performed as described in our previous work [19].

## 4. Conclusions

Nitrogen monoxide (NO) has long been used mainly as a vasodilator, e.g., in neonatal pulmonary hypertension [38]. Organic nitrates such as isosorbide-5-mononitrate and isosorbide dinitrate, acting as NO donors, are used in coronary artery disease [39]. Naproxcinod, the nitrooxybutoxy ester of naproxen, is one of the “cyclooxygenase inhibiting nitric oxide donors (CINODs)” aiming to reduce inflammation with concomitant vasodilatation and platelet inhibition. However, the Food and Drug Administration did not approve naproxcinod in 2010. Nicorandil (2-[(pyridin-3-ylcarbonyl)amino]ethyl nitrate), another nitric ester, is an anti-anginal agent acting both as a NO donor and as an ATP-sensitive potassium channel opener [40].

In this research, amino acid-conjugated derivatives of ibuprofen and naproxen possessing a nitric ester group were studied. A combination of in vitro, in vivo, and in silico relevant experiments was applied to examine their biological activities. It was found that they are able to reduce acute inflammation more effectively than the parent drugs. They could inhibit cyclooxygenases and act as hypolipidemic agents. In particular, compound **5** reduced inflammation by 73%, inhibited cyclooxygenase-2 (COX-2) by 95%, could bind to the active site of COX-2, and the binding energy was as low as the specific COX-2 inhibitor SC-5580. Additionally, it reduced lipidemic indices (total cholesterol, triglycerides, and LDL-cholesterol) by 55–60% in hyperlipidemic rats. It is also indicated that the 2-hydroxyethyl esters, considered as metabolites of these compounds after NO liberation, may still offer anti-inflammatory activity, thus prolonging the duration of their anti-inflammatory action. A limitation of the research could be that the number of compounds may not be enough to confirm a mechanism of activities at a cellular level. However, the above evidence may be further utilized either for testing other relative compounds for verification or for further molecular design, varying the length of the nitrooxy-side chain and/or the kind of the NO-liberating group, resulting in NO liberating multi-functional compounds for conditions implicating inflammation and lipid derangement.

## Figures and Tables

**Table 1:** Effect of compounds on the activity of cyclooxygenases (COX-1 and-2).

Compound	COX-1 % Inhibition	COX-2 % Inhibition
Ibuprofen	68	46
Naproxen	34	17
**5**	67	95
**6**	34	37
**7**	47	27
**8**	58	23
**9**	73	17

Determinations were carried out three times. SD values lie between 2 and 4% of the mean value.

**Table 2:** Effect of synthesized compounds, ibuprofen, naproxen, and NDGA on lipoxygenase.

Compound	IC_50_ (μM) *	Compound	IC_50_ (μM) *
Ibuprofen	200	**7**	51
Naproxen	218	**8**	184
**5**	78	NDGA	1.3
**6**	264		

*: After 7 min of incubation. NDGA: nordihydroguaiaretic acid. Determinations were performed at least in triplicate. Standard Deviation (SD) values are accepted if they are between 2 and 4% of the mean value.

**Table 3:** In vitro nitrogen monoxide release.

Compound (μΜ)	NO Release (μΜ)			
	5	6	7	8
500	163.7	97.1	79.5	95.5
250	89.4	50.4	39.5	51.5
125	42.3	23.8	18.6	24.8
62.5	20.8	12.0	9.6	11.7
31.25	8.5	5.3	5.4	6.3

All determinations (using the Griess reagent) were performed at least in triplicate, and the Standard Deviation was always within ±10% of the mean value. NO release from the reference NO donor *S*-nitroso-*N*-acetylpenicillamine (SNAP) (100 μM) was found to be 56.3%.

**Table 4:** Effect of the tested compounds as well as of ibuprofen and naproxen (0.15 mmol/kg of body weight *i.p*.) on paw edema produced by the *i.d*. administration of carrageenan to rats.

Compound	% Edema Inhibition
Ibuprofen	36 **
Naproxen	11 *
**1**	14 *
**2**	20 *
**5**	73 ***
**6**	66 ***
**7**	36**
**8**	44 **
**9**	46 * (#)

The anti-inflammatory activity is defined, in percentage, as inhibition of edema in comparison to controls which were administered carrageenan only. Each value is the mean obtained from six rats. Significant difference from carrageenan control: * *p* < 0.01, ** *p* < 0.001, *** *p* < 0.001, # *p* = 0.961 (non significant) compared with **7** (Student’s *t* test, unpaired).

**Table 5:** Effect of the tested compounds (150 μmol/kg *i.p*.) on plasma lipid levels in tyloxapol-induced hyperlipidemia.

Compound	Percent Reduction		
	TC	TG	LDL
**5**	56.7	54.8	59.8
**6**	58.4	60.4	58.7
Simvastatin	73.0	-	70.0
Ibuprofen (300 μmol/kg)	41.0	38.0	41.6

TC: total cholesterol; TG: triglycerides; LDL: low-density lipoprotein cholesterol. Groups of six rats were used. In all cases, *p* < 0.0001 (extremely significant difference from the hyperlipidemic control group, Student’s *t*-test, unpaired).

**Table 6:** Docking-assisted prediction of binding parameters.

Comp.	Ovine COX-1 (PDB:1EQG)		Mus Musculus COX-2 (PDB: 1CX2)	
	Binding Free Energy (kcal/mol)	Hydrogen Bonds	Binding Free Energy (kcal/mol)	Hydrogen Bonds
**5**	−7.71	O-Tyr355, O-Ser530	−9.24	O-Arg120, O-Tyr355, O-Tyr355
Ibuprofen	−9.22	O-Arg120, O-Arg120, O-Tyr355	-	-
SC-558	-	-	−9.17	O-His90, F-Arg120, H-Arg513

SC-558: 4-[5-(4-bromophenyl)-3-(trifluoromethyl)-1H-pyrazol-1-yl] benzenesulfonamide, selective COX-2 inhibitor used as reference compound.

## Data Availability

The original contributions presented in this study are included in the article/Supplementary Material. Further inquiries can be directed to the corresponding author(s).

## Supplementary Materials

The following supporting information can be downloaded at: https://www.mdpi.com/article/10.3390/molecules30183744/s1.
