# Supplementary figures and images for: Nitrogen Monoxide Releasing Nitric Ester Derivatives of Ibuprofen and Naproxen as COX Inhibitors, Anti-Inflammatory and Hypolipidemic Compounds
Source: Molecules. 2025 Sep 15;30(18):3744. doi: 10.3390/molecules30183744 (PMC12472888; doi:10.3390/molecules30183744)

# Compound 5

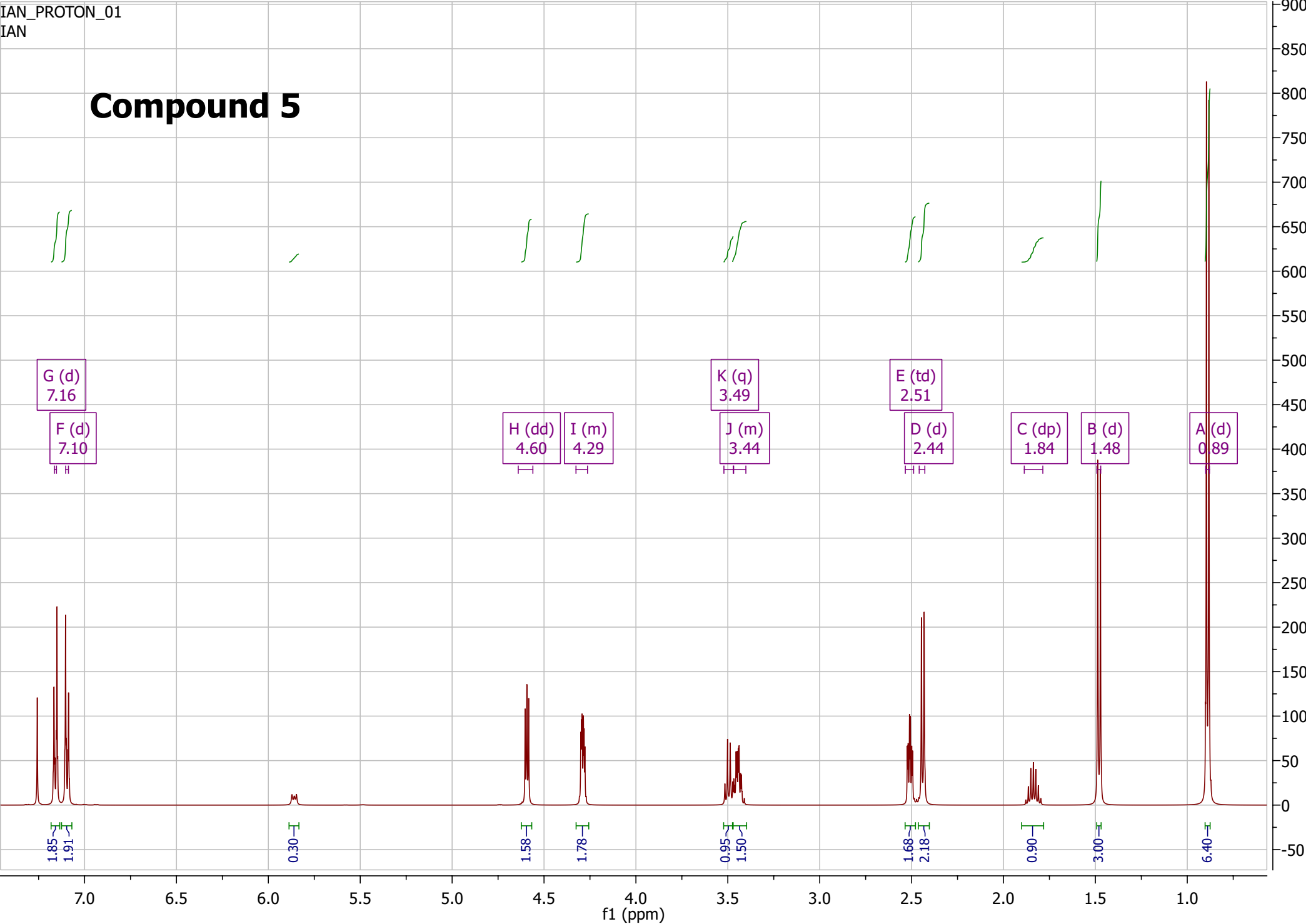

CARBON\_01  
ka\_kkka\_cdcl3

# Compound 5

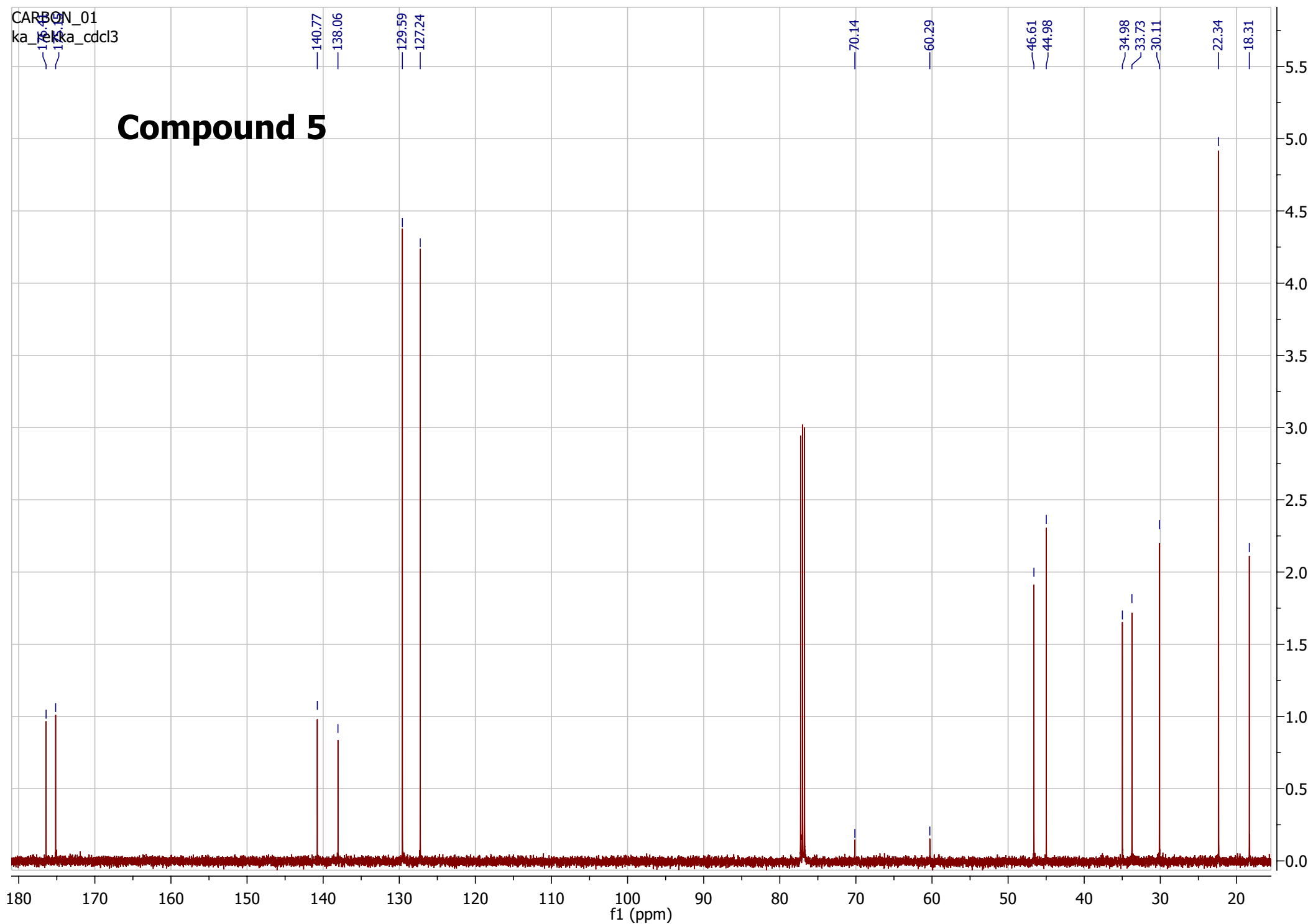

Compound 6

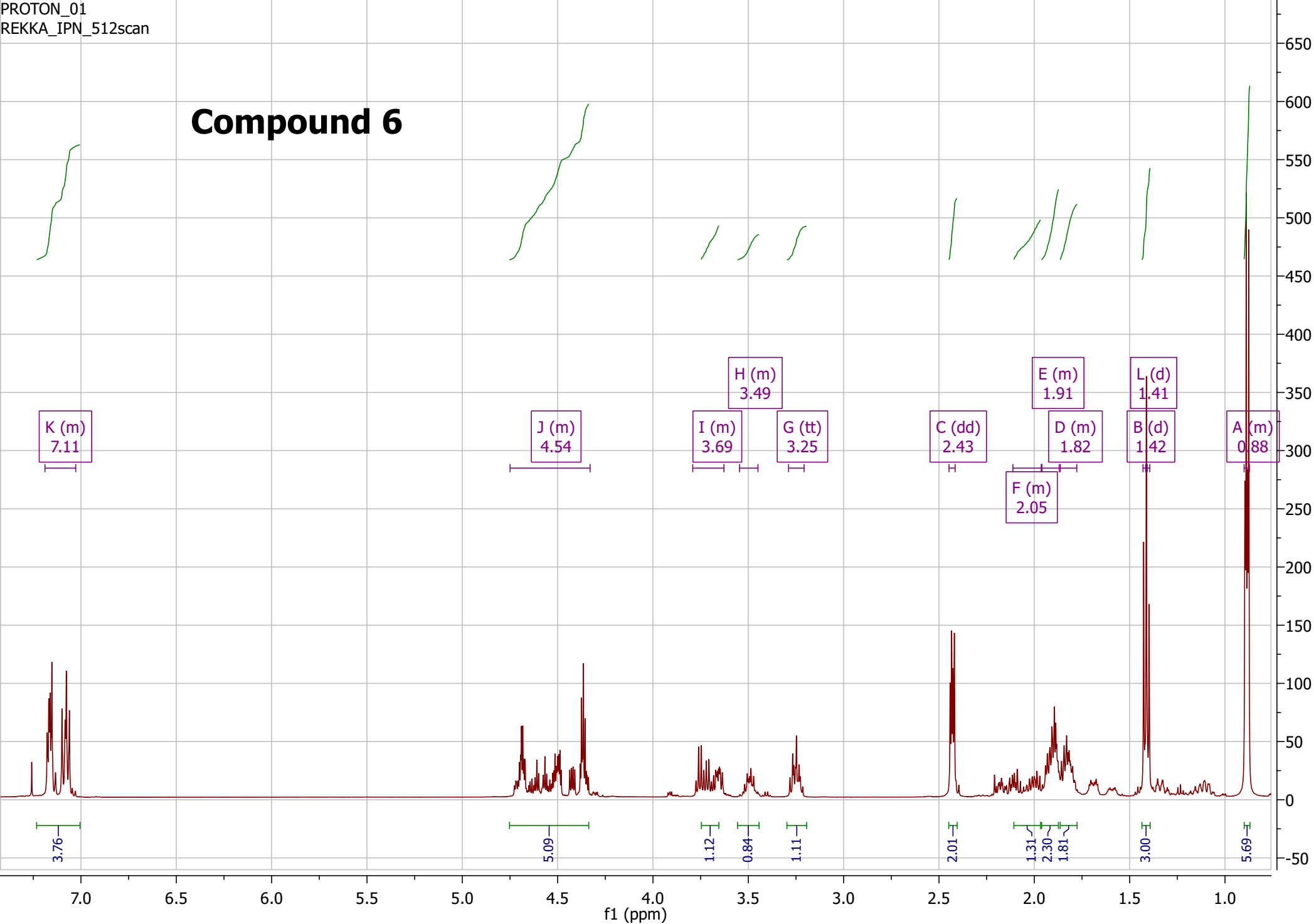

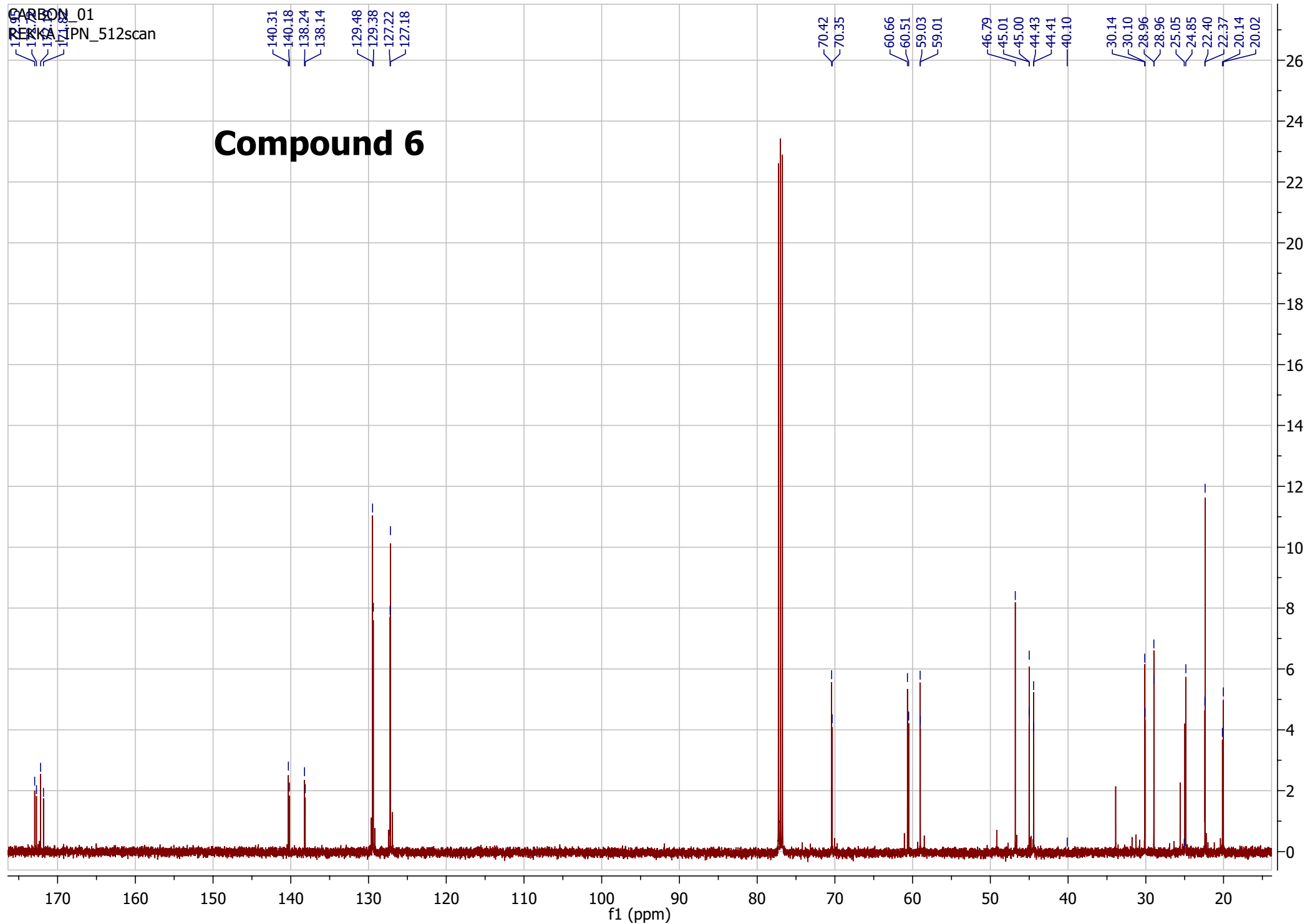

Compound 7

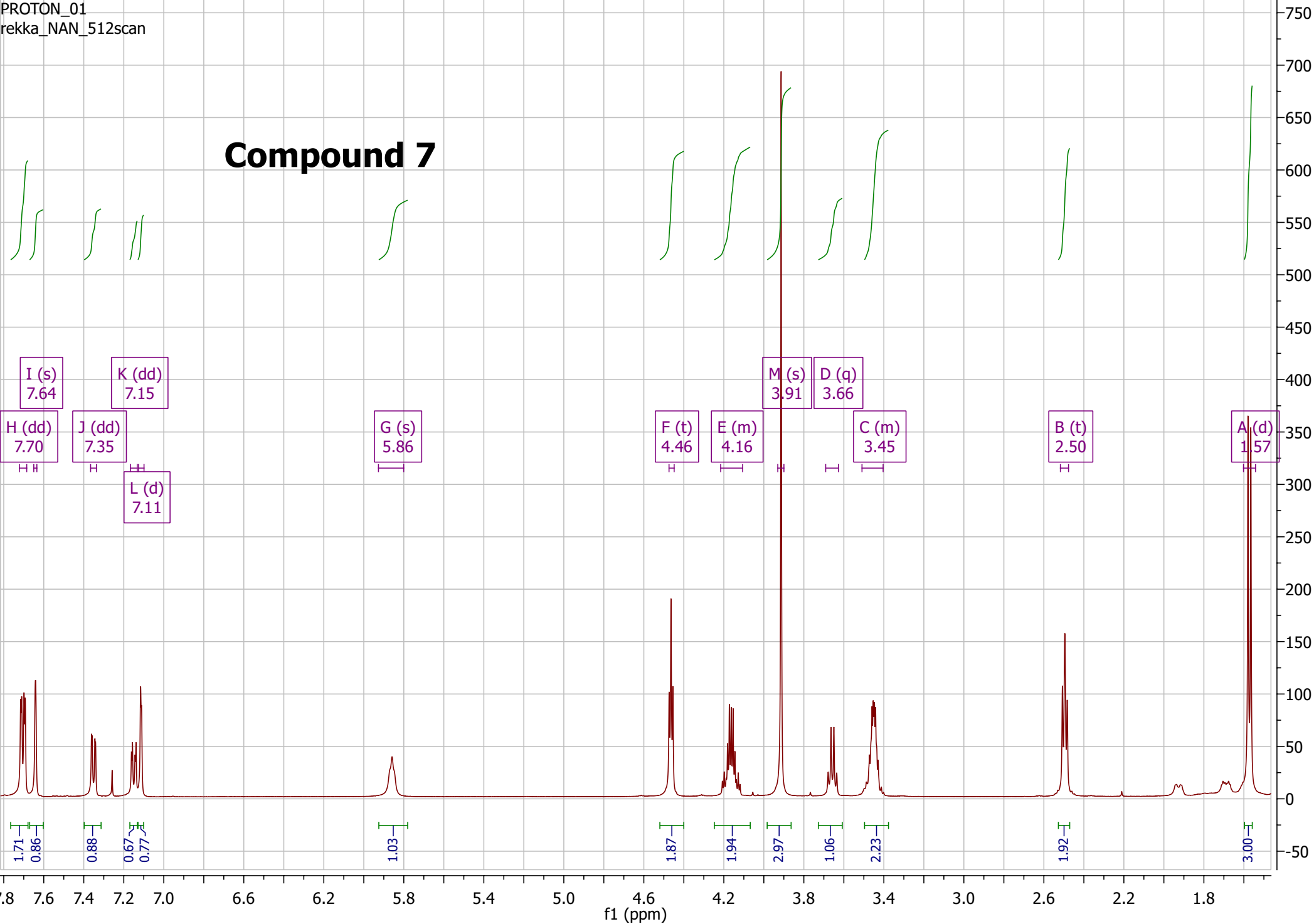

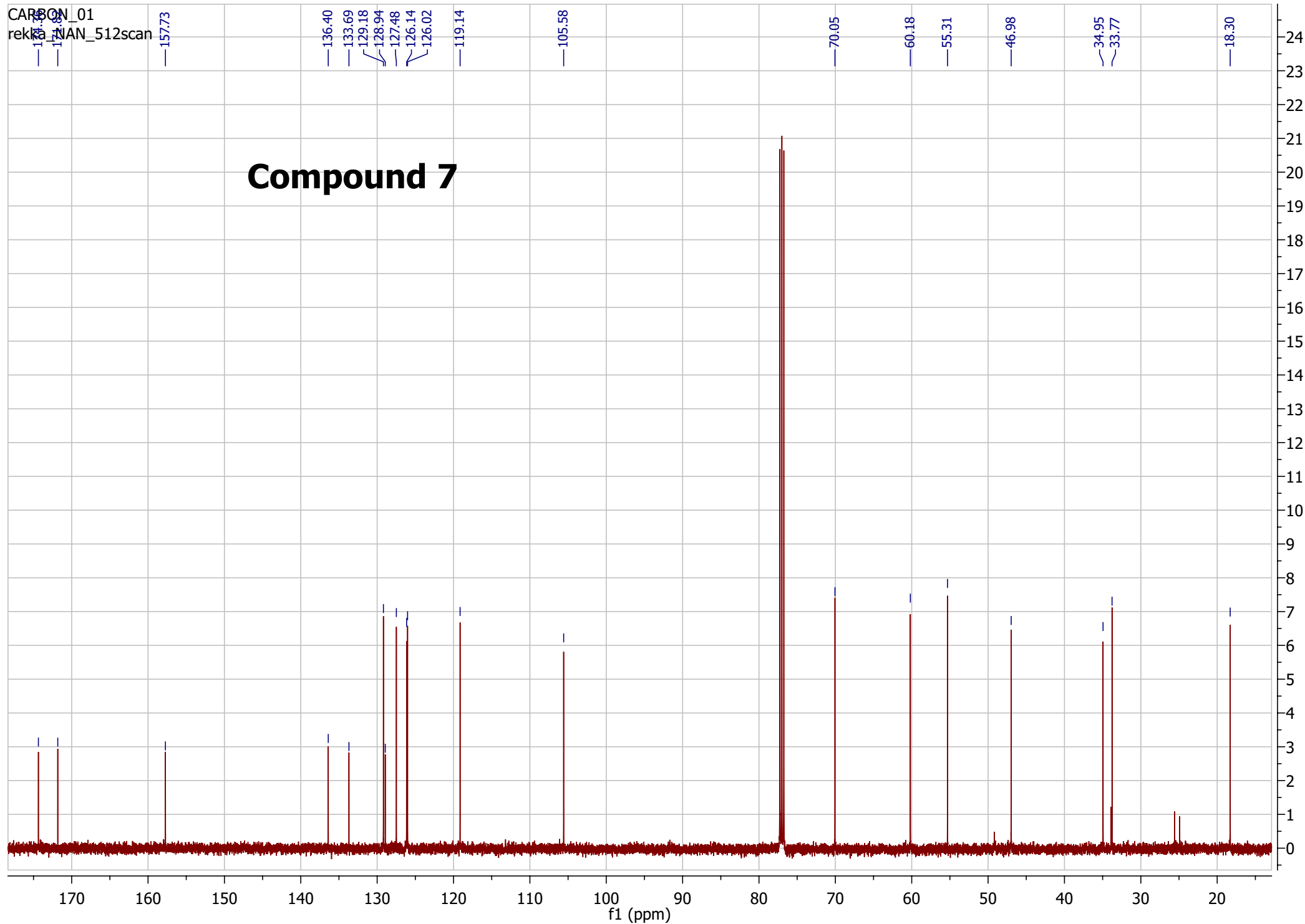

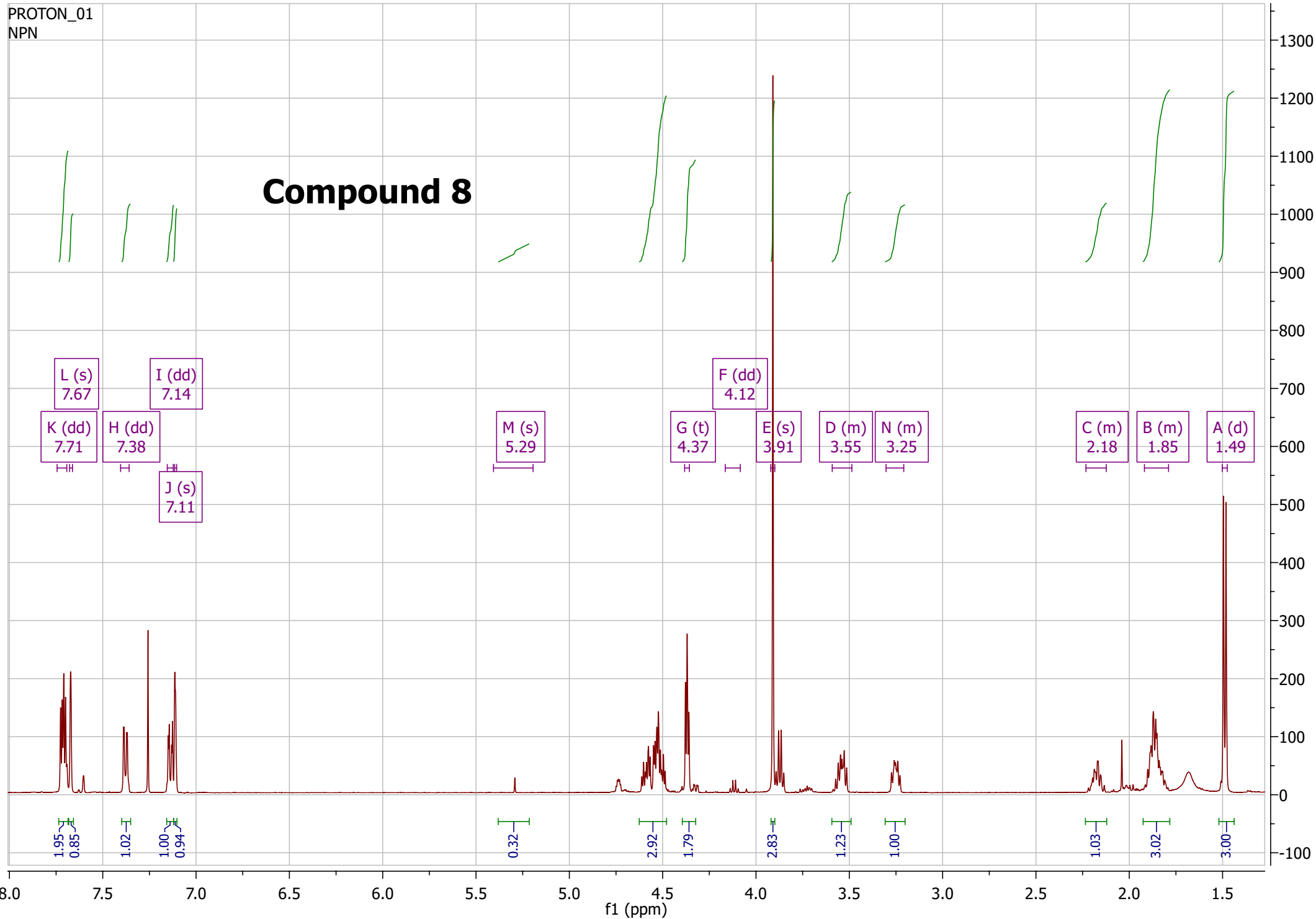

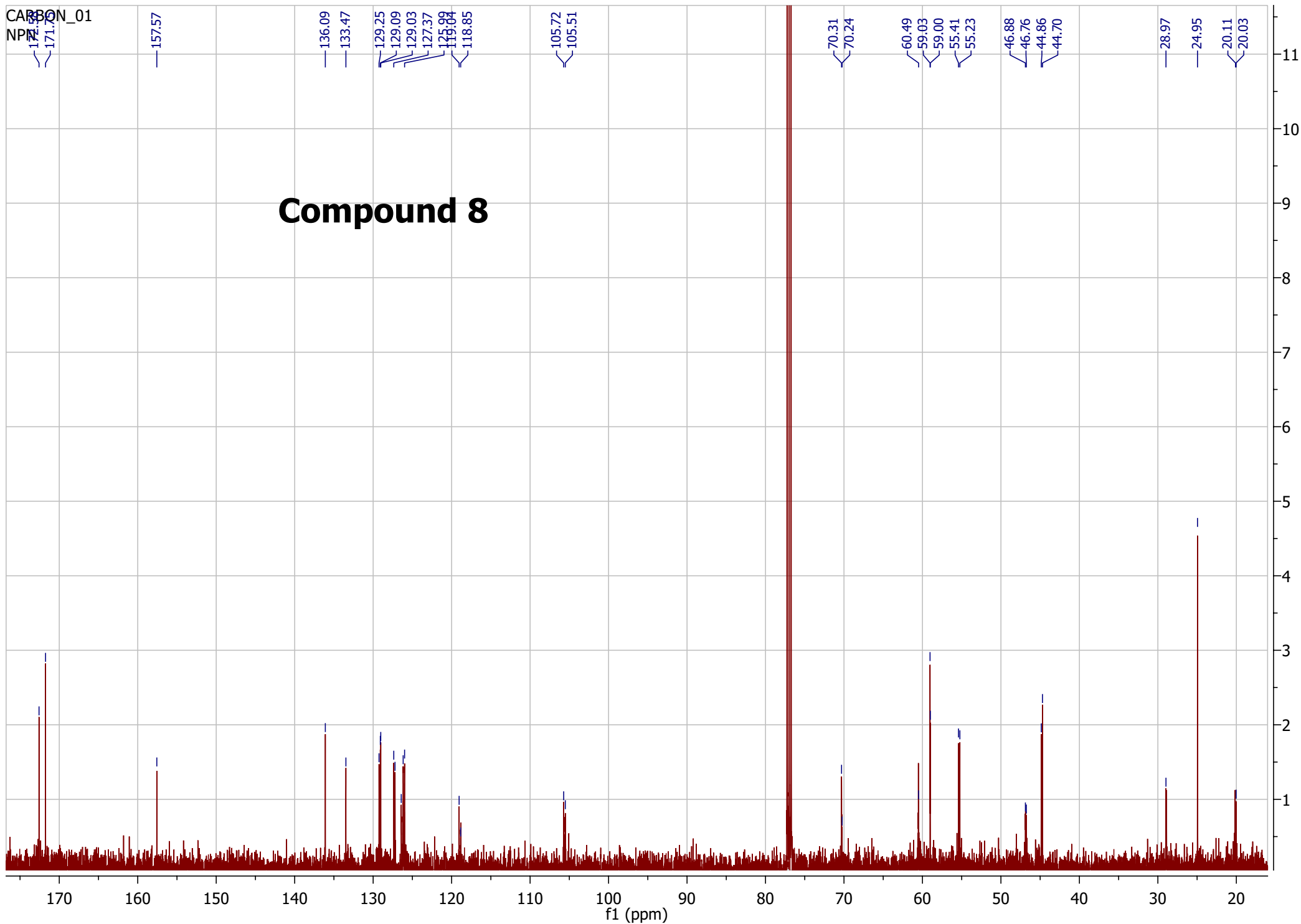

PROTON\_01  
NAD

Compound 9

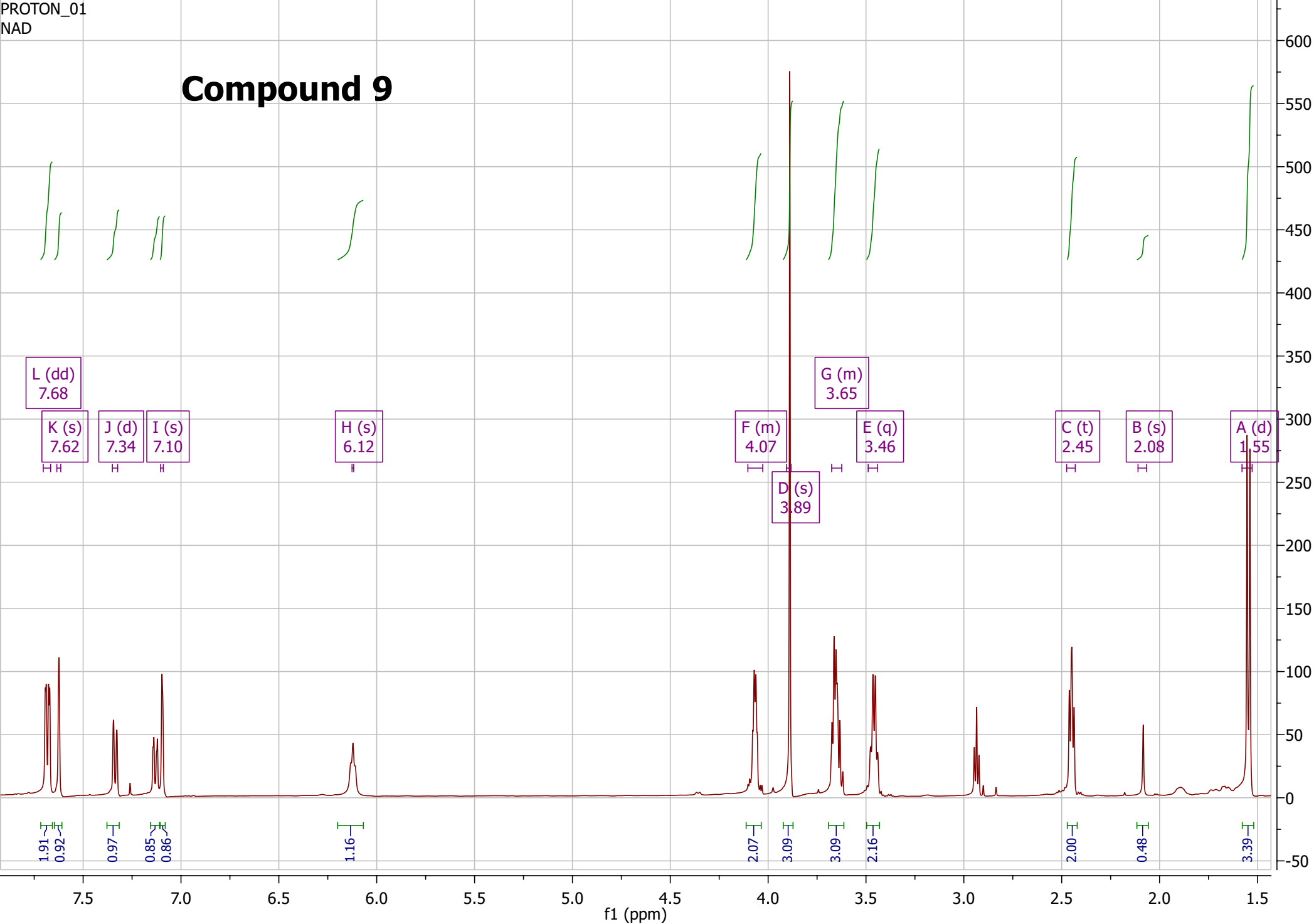

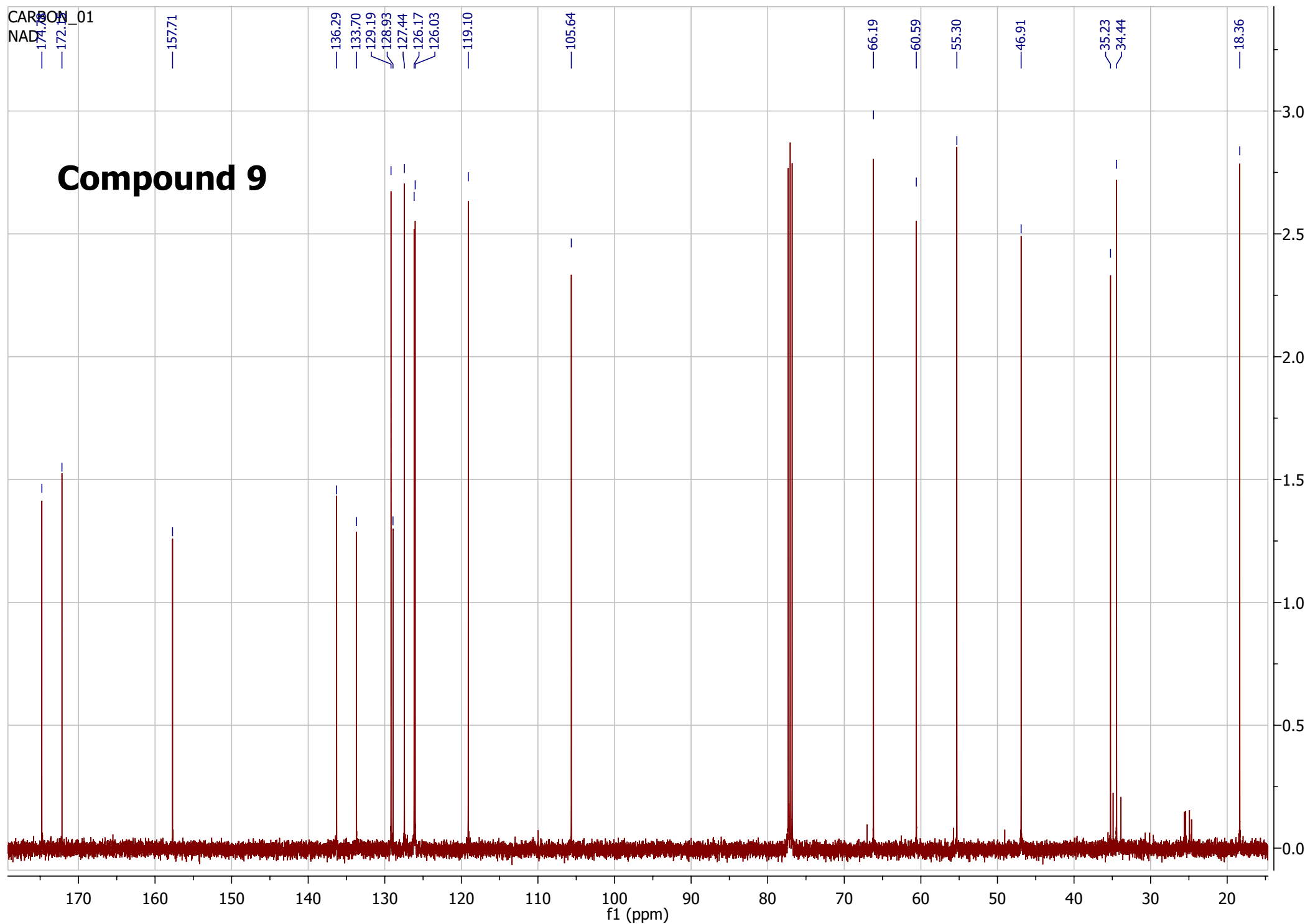

Supplement: Supplementary file 1 [file molecules-30-03744-s001.zip › molecules-3857413-supplementary.pdf]
